# Availability, prices and affordability of essential medicines in Haiti

**DOI:** 10.7189/jogh.03.020405

**Published:** 2013-12

**Authors:** Harinder Singh Chahal, Nazaire St. Fort, Lisa Bero

**Affiliations:** 1 Department of Clinical Pharmacy, University of California, San Francisco, Cal., USA; 2 Haiti Initiative, University of California, San Francisco, Cal., USA; 3 Clinical Pharmacy and Health Policy, University of California, San Francisco, Cal., USA

## Abstract

**Background:**

Haiti is the poorest country in the Western Hemisphere and faces numerous challenges, including inadequate medication access for its residents. The objective of this study was to determine the availability, prices, and affordability of essential medicines in Haiti and compare these findings to other countries.

**Methods:**

We conducted a cross–sectional nationwide survey in 2011 of availability and consumer prices of 60 essential medicines in Haiti using a standardized methodology developed by the World Health Organization and Health Action International. The survey was conducted in 163 medicine outlets in four health care sectors (public, retail, nonprofit and mixed sectors). Medicine prices were expressed as ratios relative to the International Reference Price. Affordability was calculated by comparing the costs of treatment for common conditions with the salary of the lowest paid government worker and was compared to available data from four Latin American countries.

**Results:**

For generic medicines, the availability in public, retail, nonprofit and mixed sectors was 20%, 37%, 24% and 23% of medications, respectively. Most of the available medicines were priced higher than the International Reference Price. The lowest paid government worker would need 2.5 days’ wages to treat an adult respiratory infection with generic medicines from the public sector. For treatment of common conditions with originator brands (OB) purchased from a retail pharmacy, costs were between 1.4 (anaerobic bacterial infection) and 13.7 (hyperlipidemia) days’ wages, respectively. Treatment of pediatric bacterial infections with the OB of ceftriaxone from a retail pharmacy would cost 24.6 days’ wages. Prices in Bolivia, Colombia, Mexico and Nicaragua were frequently lower for comparable medications.

**Conclusions:**

The availability of essential medicines was low and prices varied widely across all four sectors. Over 75% of Haitians live on less than US$ 2.00 /day; therefore, most medication regimens are largely unaffordable. Inclusion of essential medications on the national formulary and working with organizations responsible for importing medications into Haiti, particularly drug donation agencies, are important first steps to increasing medication access.

The World Health Organization (WHO) Essential Medicines List (EML) serves as a model for public supply and reimbursement of medicines. The list was first drafted in 1977 and expanded in 2007 to include essential medicines for children. The list highlights the most critical medicines for adult and pediatric patients [1]. Biannual revisions of the list take into account disease prevalence and the safety and efficacy of medicines, and since 2002, have adhered to rigorous standards of evidence [2]. The essential medicines concept may also be used to develop evidence–based clinical guidelines and a national medicines policy.

Data from national surveys have shown that access to essential medicines, particularly for children, is generally poor and prices can be unaffordable [3–8]. The reasons for the lack of access to essential medicines can include the absence of essential medicines policies, no regulated medicines, fragile supply systems, or out–of–pocket payments which make the medicines unaffordable. The EML can be adopted by countries according to their priority health care needs. National essential medicines lists (NEML) can be an important first step toward ensuring access to medicines since they can guide procurement, local licensing and manufacturing, and the quality use of essential medicines [9].

Haiti is the poorest country in the Western Hemisphere and, after the 2010 earthquake, has been facing significant challenges in meeting the health care needs of its residents [10]. The availability of essential medicines to address some of these needs is unknown. Haiti is a low income country with a GDP of US$ 726 per capita in 2011 [11]. About 54% of the population lives on less than US$ 1/day, and 78% live on less than US$ 2/day [12]. In 2010, Haitian life expectancy at birth was 60 years for males and 63 years for females compared with a regional average of 73 and 79 years for males and females, respectively [13]. In Haiti in 2011, mortality among children under 5 was 70 per 1000 compared to the rate of 19 per 1000 live births in the WHO Americas region [14]. The Haiti maternal mortality rate in 2010 was 350 deaths per 100 000 live births compared to a regional rate of 63 per 100 000 live births [13].

The poor health care conditions in Haiti are related to many factors including inadequate health care infrastructure, lack of health care providers and lack of health education. However, one of the least studied areas of health care in Haiti has been access to essential medicines. Many of the existing health problems facing Haiti can be treated or prevented by the use of essential medicines. Tuberculosis remains endemic and is a significant cause of mortality [15]. Malaria continues to remain a deadly problem in Haiti with a prevalence rate of 2–3% as of 2010 [16]. In 2010, diarrhea was found to be the fifth leading cause of death for Haitian children under 5, accounting for 7% of deaths [17]. PROMESS, the Program for Essential Medicines and Supply, is the central agency for the provision of essential medicines and supplies in Haiti. Under PAHO/WHO technical and managerial leadership since 1992, PROMESS is the main storage and distribution center that coordinates the efforts and contributions of international partners to improve access to essential medicines [18].

The objective of this study was to measure the availability, prices, and affordability of essential medicines in Haiti and compare these findings to other countries. These data provide evidence to guide policy ensuring that all Haitians have access to and the ability to afford life–saving medications.

## METHODS

### Study design and site

The cross–sectional survey of medicine availability and prices was conducted according to the WHO and Health Action International (WHO/HAI) methodology to facilitate comparisons with other countries. The survey of was conducted nationwide, in all ten regions of Haiti, in August 2011.

### Selection of medicine outlets

Sampling of medicine outlets was conducted according to the WHO/HAI methodology, which has been shown through a recent validation study to yield a nationally representative sample [19]. Lists of health facilities and retail pharmacies were provided by the Ministry of Health and the Regional Health Departments for each survey region. Within each region, the main public hospital was selected. Then, four to five medicine dispensing outlets (eg, hospital out–patient medicine outlets, dispensaries) were selected from those within a 4–hour drive from the main hospital in each sector. Data on 59 medicines and one device was collected from 54 public, 35 private (retail), 39 nonprofit, and 35 mixed sector medicine outlets. Mixed sector contains outlets that are managed in a collaborative partnership between the Ministry of Health and a nonprofit group.

### Selection of medicines

The WHO/HAI methodology specifies a core list of 14 global medicines and 16 regional medicines commonly used in the treatment of a range of chronic and acute conditions [19]. To facilitate international comparisons, the methodology also includes the specific dosage form and strength to be surveyed for each medicine.

In Haiti, all medicines from the WHO/HAI core lists were included in the survey [20]. An additional 28 medicines and 1 device identified as high priority essential medicines for children by the WHO Better Medicines for Children project were also included in the survey [21]. Enalapril 5mg, from the Haiti NEML [22] was also added to the survey to be evaluated alongside the Enalapril 10 mg from the regional core list. The list of survey medicines is provided in Online Supplementary Document(Online Supplementary Document), Table w1.

For each medicine in the survey, data were collected for the originator brand, highest priced generic equivalent, and lowest priced generic at each facility.

### Data collection

The survey team consisted of 14 data collectors; 5 student pharmacists from University of California, San Francisco – School of Pharmacy, 6 students from State University of Haiti – Faculty of Medicine and Pharmacy (UEH–FMP), and 1 physician, 1 pharmacist, and 1 alumni from UEH. All survey personnel received training in survey methodology and data collection procedures prior to data collection. As part of the training workshop, two data collection pilot tests were conducted at retail medicine outlets which did not form part of the survey sample.

Data collection took place between August 4 and August 16, 2011. Supervisors checked all forms at the end of each day of data collection, and validated the data collection process by collecting data at 20% of the medicine outlets and comparing their results with those of the data collectors. Discrepancies were rare between data collectors and supervisors and were corrected when found. When at least 50% of the targeted medicines in any given medicine outlet were not found, an attempt was made to survey an additional outlet. All outlets were included in the analysis.

### Data analysis

The availability of individual medicines is calculated as the percentage (%) of outlets where the medicine was found. Mean (average) availability was calculated for originator brands and the lowest priced generics for the basket of all 60 medications within each sector.

Data from Haiti were compared to data from Nicaragua, Mexico, Colombia, and Bolivia as these were the countries in the Americas region with available, recent essential medicines surveys. To facilitate cross–country comparisons, medicine patient prices obtained during the survey are expressed as ratios relative to a standard set of international reference prices: Median Price Ration (MPR) = Median Local Unit Price / International Reference Unit Price.

Thus, a median price ratio of 2 would mean that the local medicine price is twice that of the International Reference Price. Median price ratios were calculated only for medicines with price data from at least 4 medicine outlets. The exchange rate used to calculate median price ratios was 1 US$ = 39.6862 Gourdes; the commercial “buy” rate on the first day of data collection [23].

The 2010 International Drug Price Indicator Guide was used to determine the reference prices [24]. These reference prices are the medians of recent procurement prices offered by for–profit and not–for–profit suppliers to international not–for–profit agencies for generic products.

The affordability of treating seven common conditions for adults and two common conditions for children was assessed by comparing the total cost of the lowest priced generic medicines prescribed at a standard dose to the daily wage of the lowest paid unskilled government worker of US$ 5.04 [25]. For acute conditions, treatment duration was defined as a full course of therapy, while for chronic diseases, the affordability of a 30 days’ supply of medicines was determined.

This study was reviewed by University of California, San Francisco, Committee on Human Research, and declared not to fit the definition of human research (exempt), reference number 11–06271.

## RESULTS

### Availability

*Availability of medicines in the public, private, nonprofit and mixed sectors.* Table 1 shows that the availability of lowest priced generic essential medicines varied by medicine, but was low across all sectors of health care. The mean availability of lowest priced generic medicines in the public, retail, nonprofit, and mixed sectors was 20%, 37%, 24% and 23% of medications, respectively. Originator brand availability was even lower across all sectors: public (2%), retail (5%), nonprofit (2%), and mixed (1.5%). Highest priced generics were found only in the private sector. The vast majority of outlets in all other sectors carried only one generic product per medicine. Therefore, there was insufficient data to make any comparisons to highest priced generics.

**Table 1:** Availability of lowest priced generic formulation of each medication

	Percent (%) of outlets where medicine was found			
**Medicine name**	**Public sector (n = 54)**	**Retail sector** **(n = 35)**	**Nonprofit sector** **(n = 39)**	**Mixed sector** **(n = 35)**
**Amitriptyline**	1.9	11.4	7.7	2.9
**Amlodipine**	5.6	68.6	28.2	5.7
**Amoxicillin**	63	97.1	89.7	57.1
**Amoxicillin Dispersible Tab**	14.8	5.7	23.1	8.6
**Amoxicillin suspension**	50.0	51.4	46.2	48.6
**Amoxicillin Suspension 125 mg/5 ml**	68.5	97.1	56.4	80
**Amoxicillin/Clavulanic Dispersible tab**	0	2.9	0	0
**Amoxicillin/Clavulanic Suspension**	0	8.6	0	2.9
**Atenolol**	22.2	74.3	38.5	42.9
**Atorvastatin**	0	14.3	0	0
**Azithromycin**	9.3	65.7	15.4	11.4
**Beclometasone Inhaler (100 μg)**	0	0	2.6	0
**Beclometasone inhaler (250 μcg)**	0	2.9	0	0
**Benzyl Penicillin Injection**	3.7	0	2.6	5.7
**Captopril**	29.6	57.1	30.8	31.4
**Carbamazepine Chewable Tablet**	3.7	0	2.6	0
**Carbamazepine Suspension**	0	0	0	0
**Ceftriaxone injection (1 g/vial)**	51.9	71.4	51.3	51.4
**Ceftriaxone injection (500 mg/vial)**	7.4	2.9	12.8	2.9
**Chloramphenicol Injection (1g/vial)**	11.1	22.9	15.4	5.7
**Chloroquine**	55.6	77.1	71.8	62.9
**Ciprofloxacin**	57.4	91.4	76.9	77.1
**Clonazepam**	0	5.7	0	0
**Clotrimazole topical cream**	14.8	45.7	25.6	11.4
**co–trimoxazole Dispersible Tablet**	9.3	2.9	15.4	20
**Co–trimoxazole suspension**	51.9	62.9	64.1	71.4
**Diazepam**	25.9	45.7	30.8	40
**Diazepam Rectal Solution**	0	0	0	0
**Diclofenac**	46.3	85.7	43.6	42.9
**Enalapril**	35.2	85.7	41	37.1
**Enalapril (5mg)**	11.1	82.9	15.4	25.7
**Ferrous Salt Suspension**	3.7	5.7	0	8.6
**Fluoxetine**	5.6	14.3	0	2.9
**Furosemide**	38.9	77.1	48.7	60
**Gentamycin Injection**	5.6	0	2.6	0
**Glibenclamide**	29.6	80	41	54.3
**Hydrochlorothiazide**	38.9	65.7	35.9	34.3
**Ibuprofen (200 mg)**	24.1	31.4	56.4	40
**Ibuprofen (400 mg)**	66.7	82.9	61.5	80
**Isoniazid**	0	0	0	0
**Metformin**	20.4	62.9	15.4	8.6
**Metronidazole**	46.3	88.6	59	48.6
**Morphine Dispersible Tablet**	0	0	5.1	0
**Morphine Oral Solution**	0	0	0	0
**Omeprazole**	40.7	97.1	46.2	34.3
**Oral Rehydration Solution (1 L)**	48.1	45.7	69.2	80
**Oral Rehydration Solution (500 ml)**	0	0	0	5.7
**Paracetamol suspension (120 mg/5 ml or 125 mg/5 ml)**	48.1	77.1	51.3	60
**Phenobarbital Injection**	0	0	0	0
**Phenobarbital Oral Liquid**	3.7	8.6	0	0
**Phenytoin**	9.3	17.1	25.6	11.4
**Phenytoin Chewable Tablet**	0	0	0	0
**Phenytoin Suspension**	0	0	0	0
**Procaine Penicillin Injection**	3.7	8.6	0	2.9
**Ranitidine**	24.1	80.0	33.3	51.4
**Salbutamol inhaler**	25.9	85.7	35.9	37.1
**Simvastatin**	1.9	57.1	2.6	0
**Spacer (for Inhalers)**	0	0	2.6	0
**Vitamin A**	25.9	8.6	23.1	14.3
**Zinc Dispersible Tablet**	7.4	2.9	5.1	11.4

*International comparisons of private sector availability.* International comparison of the availability of nine originator brands in the private sectors was possible across Haiti, Nicaragua, Mexico, Colombia, and Bolivia. Compared to other countries, Haiti had the lowest originator product availability for atenolol (11% of outlets), ciprofloxacin (6%), and diclofenac (34%). The overall availability of the nine originator products that were surveyed across all countries was 13% of medicines in Haiti, compared to 19%, 47%, 23%, and 4% in Nicaragua, Mexico, Colombia, and Bolivia, respectively.

Table 2 shows the country comparisons of the availability of 12 lowest priced generic medicines in the private sector that were surveyed in all 5 countries. The average availability of lowest priced generics in Haiti was 73% for these 12 medications, compared to 84% in Nicaragua, 48% in Mexico, 79% in Colombia, and 71% in Bolivia.

**Table 2:** Availability and median price ratios (MPR) of 12 essential lowest priced generic medications in Haiti compared to 4 neighboring countries

	Haiti		Nicaragua		Mexico		Colombia		Bolivia	
Medication (strength)	Availability (%)*	MPR	Availability (%)	MPR	Availability (%)	MPR	Availability (%)	MPR	Availability (%)	MPR
**Amitriptyline 25 mg**	11.4	13.3	51.6	11.2	0	N/A	93.2	6.4	50	8.3
**Amoxicillin 500 mg**	97.1	4.3	100	2.2	53.3	4.3	96.6	2.5	100	2.3
**Atenolol 50 mg**	74.3	15.9	61.3	4	6.7	N/A	11.9	10.1	73.3	6.15
**Captopril 25 mg**	57.1	10.5	93.5	4	86.7	5.5	96.6	1.5	0	N/A
**Ceftriaxone 1 g/vial**	71.4	5.5	90.3	3.6	73.3	6.6	49.2	3	93.3	1.1
**Ciprofloxacin 500 mg**	91.4	5.1	100	8	80	12.7	100	4.8	96.7	4.4
**Co–trimoxazole 8 + 40 mg/ml**	62.9	4.5	83.9	4.2	80	4.5	86.4	4	86.7	4
**Diclofenac 50 mg**	85.7	25.2	96.8	11.3	6.7	N/A	96.6	7.9	100	7.9
**Glibenclamide 5 mg**	80	20.4	83.9	10.4	46.7	5.2	91.5	7.3	90	13
**Omeprazole 20 mg**	97.1	4.2	96.8	6.3	80	9.3	94.9	2.2	96.7	3.3
**Salbutamol 100 μg/dose**	85.7	2.3	71	3	53.3	2.1	96.6	1.4	60	2.4
**Simvastatin 20 mg**	57.1	N/A	83.9	N/A	6.7	N/A	35.6	N/A	6.7	N/A

N/A – Not Available or Not Applicable

*Percent (%) of retail outlets with medication.

### Prices

*Consumer prices in public, private, nonprofit, and mixed sectors.* Across all four sectors, the medicines in Haiti were sold at higher prices than the international reference price. Table 3 shows that consumer prices in Haiti were closest to the international reference price for originator brands sold in the public sector. Originator brands prices in the private sector in Haiti were 35 times the international reference price.

**Table 3:** Median price ratios (MPR) of originator and lowest priced generic medicines by sector

	Type of medicine	
**Sector**	**Originator Brand**	**Lowest Priced Generic**
Public	1.6	4.8
Private	35	7
Nonprofit	N/A^	4.3
Mixed	N/A^	4.0

MPR – Median Price Ratio = median local unit price/international reference unit price, N/A = insufficient data available for calculation.

Table w2 in Online Supplementary Document(Online Supplementary Document) shows median price ratios for selected lowest priced generic medications, by sector.

Medicines were not priced consistently in relation to their international reference price. In the public sector, half of the lowest priced generic medicines were priced at 3.4 (25^th^ percentile) to 9.0 (75^th^ percentile) times their international reference price. In the retail sector, half of the originator brand medicines were priced at 11.2 (25^th^ percentile) to 47.4 (75^th^ percentile) times their international reference price and half of the lowest priced generic medicines were priced at 4.4 (25^th^ percentile) to 14.3 (75^th^ percentile) times their international reference price. In the nonprofit sector, half of the lowest priced generic medicines were priced at 3.3 (25^th^ percentile) to 10.2 (75^th^ percentile) times their international reference price and finally in the mixed sector, half of the lowest priced generic medicines were priced at 3.1 (25^th^ percentile) to 6.9 (75^th^ days’ percentile) times their international reference price.

*International comparisons of private sector prices.* As shown in Table 2, most of the 12 lowest priced generic medications were sold at higher prices in Haiti compared to Nicaragua, Mexico, Colombia, and Bolivia. On average, these medications were sold in Haiti at 10 times the international reference price, compared to seven times the international reference price in Nicaragua, 6 times the international reference price in Mexico and Colombia, and 5 times the international reference price in Bolivia.

### Affordability

*Affordability of medicines to treat common conditions.* Table 4 shows that most of the lowest priced generics needed to treat 10 common uncomplicated conditions cost less than a day’s wage in the public sector. Treatments costing over a day’s wage include diabetes with metformin 850 mg (1.5 days’ wages) and hypertension with captopril 25 mg (1.2 days’ wages). However, given the low availability of medicines in the public sector, many patients must purchase medicines from the private sector.

**Table 4:** Number of days’ wages needed for the lowest paid Haitian government worker to purchase standard treatments for adults and children in Haiti

For Adults (daily wage: 200 HTG (US$ 5.04/day)					
**Disease condition and ‘standard’ treatment**		**Day’s wages to pay for treatment**			
**Condition, drug name, strength, dosage form**	**Treatment schedule**	**Lowest priced generic – public sector**	**Lowest priced generic – private sector**	**Lowest priced generic – nonprofit sector**	**Lowest priced generic – mixed sector**
**Asthma**					
Salbutamol 100 μg/dose inhaler	1 inhaler of 200 doses	0.7	0.8	0.6	08
**Diabetes**					
Glibenclamide 5 mg cap/tab	1 cap/tab ×2 × 30 days = 60	0.8	0.8	0.5	0.6
Metformin 850 mg cap/tab	1 cap/tab ×2 × 30 days = 60	1.5	1.7	n/a	N/A
**Hypertension**					
Atenolol 50 mg cap/tab	1 cap/tab ×30 days = 30	0.4	0.9	0.6	0.8
Captopril 25 mg cap/tab	1 cap/tab ×2 30 days = 60	1.2	1.5	1.5	0.9
Amlodipine 5 mg cap/tab	1 cap/tab ×30 days = 30	N/A	0.8	0.5	N/A
**Hyperlipidemia**					
Simvastatin 20 mg cap/tab	1 cap/tab ×30 days = 30	N/A	2.1	N/A	N/A
Atorvastatin 10 mg cap/tab	1 cap/tab ×30 days = 30	N/A	2.6	N/A	N/A
**Bacterial infection**					
Ciprofloxacin 500 mg cap/tab	1 cap/tab ×2 for 7 days = 14	0.4	0.4	0.4	0.4
Amoxicillin 500 mg cap/tab	1 cap/tab ×3 for 7 days = 21	0.3	0.5	0.4	0.4
Ceftriaxone 1 g/vial injection	1 vial ×7 days = 7 vials	2.5	5.3	4.2	3.5
**Anxiety**					
Diazepam 5 mg cap/tab	1 cap/tab ×7 days = 7	0.2	0.2	0.1	0.1
**Arthritis**					
Diclofenac 50 mg cap/tab	1 cap/tab ×2 × 30 days = 60	0.6	1.3	0.6	0.5
**Ulcer**					
Omeprazole 20 mg cap/tab	1 cap/tab ×30 days = 30	0.8	0.8	0.7	0.5
Ranitidine 150 mg cap/tab	1 cap/tab ×2 × 30 days = 60	0.9	1.4	0.9	0.8
**For Children**					
**Bacterial infection**					
Amoxicillin Suspension 125 mg/5 mL	Child up to 10 years: 125 mg ( = 5 ml) × 3 × 7 days = 105 ml	0.3	0.3	0.3	0.3
Amoxicillin Suspension 250 mg/5 mL	Child over 10 years: 250 mg ( = 5 ml) × 3 × 7 days = 105 ml	0.3	0.4	0.3	0.3
Co–trimoxazole 8 + 40 mg/ml suspension	5 ml twice a day for 7 days = 70 ml	0.2	0.3	0.2	0.4
**Pain/inflammation**					
Paracetamol 24 mg/ml suspension	5-year-old child: 15 mg/kg ×20 kg ×4 × 3 days= 3600 mg ( = 150 mL)*	0.5	0.6	0.4	0.5

*Weight of average 5-year-old child = 20 kg (Centers for Disease Control and Prevention, United States).

Table 4 shows that in the private sector, the affordability of the lowest priced generics varies from 0.2 to 5.3 days' wages. Treatments that cost more than one day’s wage include diabetes with metformin 850 mg (1.7 days’ wage), hypertension with captopril 25 mg (1.5 days’ wage), and hyperlipidemia with simvastatin 20 mg (2.1 days’ wage). Treatment of respiratory infection with ceftriaxone 1g/vial cost 5.3 days’ wage. The most affordable standard treatments were those for treating chronic conditions such as asthma with salbutamol 100mcg (0.8 days’ wage) and diabetes with glibenclamide 5 mg (0.8 day’s wage). The most affordable standard treatments were those for treating acute conditions like respiratory infection with ciprofloxacin 500 mg and amoxicillin 500 mg (0.4 and 0.5 days’ wage, respectively).

When originator brands are prescribed and dispensed in the private sector, several treatments cost well over one day’s wage. Treatment of respiratory infection with ceftriaxone 500 mg and ceftriaxone 1g costs 24.6 and 28.0 days’ wages, respectively, while treating arthritis with diclofenac 50 mg costs 9.0 days’ wages.

We calculated the number of days’ wages required to treat a family with 3 chronic conditions. To treat a mother’s diabetes with 30 days of metformin, 1.5 days’ wages are required. In addition, 1.2 days’ wages are required to treat the father’s hypertension with captopril for 30 days’ and 0.7 days’ wages are required to treat the child’s asthma. Thus, 3.4 days’ wages, or US$ 17.14 per month, are required for the family.

**Figure 1:**
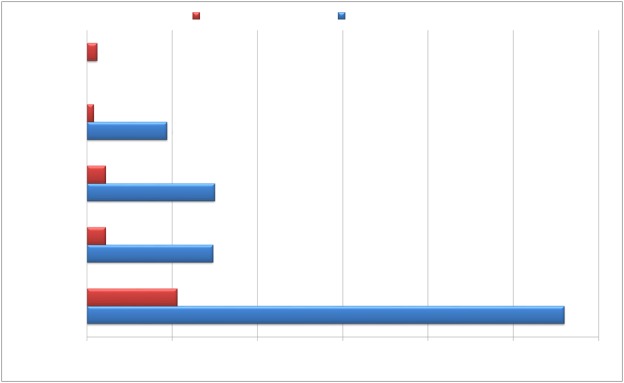
Median price ratios for treatment for adult respiratory infection with ceftriaxone 1 g/vial injection purchased in the private sector in five countries.

*International comparisons of private sector consumer prices of medicines to treat common conditions.* Data were available to compare the median price ratios of treatment for adult respiratory infection with ceftriaxone 1 g/vial injection purchased in the private sector. Figure 1 shows that with either generic or originator brand formulations, the cost of the treatment in Haiti significantly exceeds that of the comparator countries.

## DISCUSSION

The availability of essential medicines for adults and children is poor across all sectors of health care in Haiti. Generic equivalents were the predominant product type available in all outlets, across all sectors. Prices varied widely across sectors and medicines for the treatment of common conditions are not affordable for the majority of Haitians. These findings are consistent with studies of the availability and affordability of essential medicines in other countries [4,26–29].

A significant number of medicines were not found in any of the outlets – 16 medicines in the public sector, 13 in the retail sector, 15 in the nonprofit sector, and 19 in the mixed sector. Ten medicines were not found in any outlet in the public, nonprofit, and the mixed sectors. Eight of these 10 medications are used for management of pediatric conditions. Among the medicines that were lacking for pediatric use were phenytoin suspension and diazepam rectal solution for treatment of epileptic disorders, oral rehydration solution packets and amoxicillin/clavulanic acid dispersible tablets for bacterial infections.

Morphine formulations were not expected to be available at primary or clinic level of care or in retail settings. However, the surveyed formulations of morphine were also not found at secondary level regional hospitals or tertiary level hospitals. Although not surveyed, morphine in injection form was found only at the tertiary level public and nonprofit hospitals. The observed low availability may be due to the regulations placed on importation and/or manufacturing of opioid analgesics by the Haitian government [30]. Opioid availability and use has also been observed to be low after natural disasters [31]. Haiti has one of the lowest reported uses of opioid analgesics in Latin America and worldwide [30,32]. The low use of opioids for pain control may stem from the need for additional medical knowledge and biases that may exist for the treatment of pain with opioid based medications [30]. Furthermore, a comparative study found that the cost of opioids in developing countries was higher than in developed countries, which can limit availability and affordability in countries such as Haiti [33]. A recent WHO study indicated a strong positive correlation between development of a country and adequate access to opioids [30].

The prices of essential medicines in Haiti were considerably higher than the international reference price and there was a notable variability in prices across outlets in all health care sectors. The variability observed between outlets may have been the result of low market competition (as is the case outside of the capital city), the absence of price regulations on pharmaceutical products throughout the country, or differences in procurement and/or variability in price mark–ups throughout the distribution chain and in the areas surveyed. Further investigation of medicine pricing components is warranted.

In the public sector, the affordability of lowest priced generics was good for most conditions, with standard treatment costing up to one day’s wage. However, low public sector availability may force some patients to purchase higher priced medicines from the private sector. In the private sector, some of the treatments, such as those for diabetes, hyperlipidemia, and hypertension, cost close to the daily wage of the lowest paid government worker, even when lowest priced generics are used. The majority of standard treatments were much less affordable when originator brands were purchased in the private sector.

Most Haitians earn much less than the lowest government wage, so even treatments which appear affordable were too costly for the poorest segments of the population. Given that 54% of the population is living below the international poverty line of less than US$ 1/day and 78% of the population in Haiti lives on less than US$ 2/day, essential medicines are financially out–of–reach for a substantial number of people [12].

Although treatment for some acute conditions such as bacterial infections may be affordable, the ongoing costs of treating chronic illnesses such as diabetes, hypertension, and hyperlipidemia may be insurmountable for many patients in Haiti [4,28]. According to the Pan American Health Organization (PAHO) the burden of chronic diseases in Haiti is high; a 2010 study in Port–au–Prince metropolitan area found hypertension prevalence of 48.7% and 46.5% in men and women, respectively [16]. The 2010 WHO report on noncommunicable diseases estimated the prevalence of overweight and obesity in Haiti at 32%, which increases the risks for chronic conditions such as cardiovascular diseases and diabetes [34]. The situation is further complicated by the observed lower availability of medications for treatment of chronic diseases vs acute conditions [26,28]. The monthly cost of long–term management of multiple chronic illnesses can exceed several days’ wages of the lowest paid government employee. As shown in our example of a family of 3 requiring medicines for asthma, diabetes, and hypertension, the cost is US$ 17.14 per month and 54% of the Haitian population lives on US$ 30 per month. In addition, treatment costs were for medicines only and did not include the costs of consultation and diagnostic tests that place additional financial burden on patients [35,36].

Similar to adult medications, the availability of child–specific generic medicines far exceeded that of originator products across all sectors. Even so, most outlets only carried 3% to 50% of children’s essential medications. This is consistent with a recent study on the availability of children’s mediations conducted in 14 African countries, where availability of medications ranged from 15–75% of outlets, rarely exceeding 50% availability in any given outlet [8].

The affordability of treatments for children is no better than the availability. The leading causes of death of children under five in Haiti are diarrheal diseases, respiratory infections and malnutrition, and the under–five child mortality rate in Haiti in 2011 was 70 children per 1000 [14]. Amoxicillin suspension is used to manage respiratory infections, and was the most common medication available for pediatric bacterial infections. However, the suspension was sold at, on average, three times the international reference price. Originator brand dispersible zinc tablets were widely available across sectors (except retail) for the management of diarrhea [37]. The cost of the zinc tablets was about 1.5 times the international reference price in the public sector, although zinc tablets were available free of charge in the public, nonprofit, and mixed sectors. However, oral rehydration solution (ORS) was available in only about half of the public outlets and in 80% of the mixed sector outlets; and when it was found, the ORS sold at 2.4 times the international reference price in retail outlets. It is unusual for zinc to be available more often than ORS and for it to be sold at a lower median price ratio [3,8]. It is possible that the availability of zinc in Haiti increased due to campaigns such as the one undertaken by UNICEF and its supporting partners in response to the cholera outbreak in October 2010 [38].

The comparisons with other Latin American countries suggest that the availability of lowest priced generics in Haiti is similar to that of the other countries. However, since medications in Haiti are priced higher than other countries, the affordability of those medications is much less. Further research is needed to identify the reasons for variation between different countries. Possible reasons include factors like size of the markets, capabilities of the national pharmaceutical manufacturing sector, the effect of taxes, duties, and mark–ups at national and local levels, and economic indicators. Such information would be useful for policymakers and governments in deciding what specific interventions can be made to make medicines more affordable and accessible in each country. Further studies and comparisons between high and low–income countries could also provide an evidence base for equitable or differential pricing strategies by multinational manufacturers, so that less wealthy populations can pay the same or less than wealthier countries for essential medicines.

We had suspected that the increase in medication donations after the 2010 earthquake in Haiti may have resulted in a sustained influx of essential medicines in Haiti, but this was not the case. Immediately after the earthquake, PROMESS played a key role in dispersing medicines as quickly as possible to where they were needed. However, in order for PROMESS to be effective in coordinating donations over the long term, it must have adequate staffing by pharmacists and logistics experts, as well as good communication with all donating agencies [39]. Furthermore, emergency drug donations are infrequently guided by essential medicines lists [40]. Donations that are not adherent to the WHO Guidelines for Medication Donations [41], which include the recommendation that donated medicines be essential medicines, can be more burdensome to the health care system than helpful, especially in an emergency situations [37,42–44]. Haiti is a participant in the PAHO Strategic Fund created by PAHO in 2000. Through the Strategic Fund, Haiti is eligible to receive technical assistance on how to review the supply management system and develop a coordinated procurement plan which could inform the coordination of donations [45].

The use of the WHO/HAI medicine prices survey allowed us to measure prices and availability in a reliable and standardized way in order to make valid international comparisons. A further strength of the methodology is the multiple steps taken to ensure data quality [46]. However, our study has some limitations. Data on medication availability are influenced by market fluctuations and delivery schedules. Therefore data on medication availability at a single point in time may not reflect average monthly or yearly availability of medicines at individual facilities. In addition, the reliability of median price ratios is dependent on the number of supplier prices used to determine the median international reference price of each medicine. In cases where very few supplier prices are available, or where there is no supplier price and the buyer price is used as a proxy, median price ratio results can be skewed by a particularly high or low international reference price. A further limitation is that the list of medicines surveyed does not account for the availability of alternate strengths or dosage forms, or of therapeutic alternatives. Finally, the methodology does not include informal sectors, such as markets and general stores, as the quality of the medicines found in such sectors cannot be assured.

## CONCLUSION

Although further investigation is required to obtain a more in–depth understanding of the causes and consequences of medicine availability and pricing [28], our findings show that several policies are required to make medicines more affordable and available in Haiti. First, a comprehensive assessment of the supply chain should be undertaken to identify reasons for low availability as well as areas where regulation of the procurement chain are appropriate [9,28]. Second, a routine assessment of the suppliers and the storage facilities around the country should be undertaken to ensure all essential medicines are being stocked and distributed to dispensing facilities in a timely and efficient manner [28].

The government of Haiti could require that its recently adopted National Essential Medicines list be used for purchasing and donation requests by all registered health agencies in the public, nonprofit, and mixed sectors as well nonprofit and for–profit organizations responsible for production and/or importation of medications into Haiti. PROMESS could play a key role in these coordination efforts. Restricting acceptance of mass donations to EML medicines after disasters is particularly important to avoid the use of dangerous or inappropriate medicines and their associated disposal costs [40]. As a participant in the Pan American Health Organization Strategic Fund, Haiti can receive technical support in procurement planning and programming to ensure continuous availability of essential medicines [47]. The government and partners should commit to reducing the price of the lowest priced generics across all medication dispensing sectors in Haiti. Interventions such as removal of duties and taxes on essential medicines are an option to achieve this goal [27–29,48]. An availability and pricing survey should be undertaken every 2 years to allow for continuous monitoring of impact and efficacy of any new policies put in place by government and health care partners in Haiti. Broad debate and dialogue are needed to identify how stakeholders can contribute to enhancing accessibility and affordability of essential medicines.
