# Supplementary material for: Availability, prices and affordability of essential medicines in Haiti
Source: J Glob Health. 2013 Dec;3(2):020405. doi: 10.7189/jogh.03.020405 (PMC3868824; doi:10.7189/jogh.03.020405)
Supplement: Online Supplementary Document [file jogh-03-020405-s001.pdf]

Online Supplementary Document

Chalal et al. Availability, prices and affordability of essential medicines in Haiti
Journal of Global Health 2013;2:020405

Table w1. List of surveyed medicines in Haiti, 2011

List	No.	Disease	Name	Strength	Dosage form
Global core list	1	Asthma	Salbutamol	100 mcg/dose	inhaler
	2	Diabetes	Glibenclamide	5 mg	cap/tab
	3	Cardiovascular disease	Atenolol	50 mg	cap/tab
	4	Cardiovascular disease	Captopril	25 mg	cap/tab
	5	Cardiovascular disease	Simvastatin	20 mg	cap/tab
	6	Depression	Amitriptyline	25 mg	cap/tab
	7	Infectious disease	Ciprofloxacin	500 mg	cap/tab
	8	Infectious disease	Co-trimoxazole	8+40 mg/ml	suspension
	9	Infectious disease	Amoxicillin	500 mg	cap/tab
	10	Infectious disease	Ceftriaxone	1 g/vial	injection
	11	CNS	Diazepam	5 mg	cap/tab
	12	Pain/inflammation	Diclofenac	50 mg	cap/tab
	13	Pain/inflammation	Paracetamol	24 mg/ml	syrup/susp
	14	Ulcer	Omeprazole	20 mg	cap/tab
Regional core list	15	Infectious disease	Amoxicillin suspension	50 mg/ml (250mg/5ml)	Syrup/susp
	16	Hypertension	Amlodipine	5mg	Cap/tab
	17	Hypercholesterolemia	Atorvastatin	10mg	Cap/tab
	18	Infectious disease	Azithromycicn	500mg	Cap/tab
	19	Asthma	Beclometasone inhaler	250mcg/dose	Inhaler
	20	Anxiety	Clonazepam	2 mg	cap/tab
	21	Fungal Infection (topical)	Clotrimazole topical cream	1%	cream
	22	Hypertension	Enalapril	10 mg	cap/tab
	23	Depression	Fluoxetine	20 mg	cap/tab
	24	Hypertension	Furosemide	40 mg	cap/tab
	25	Hypertension	Hydrochlorothiazide	25 mg	cap/tab
	26	Pain/inflammation	Ibuprofen	400 mg	cap/tab
	27	Diabetes	Metformin	850 mg	cap/tab
	28	Infectious disease	Metronidazole	500 mg	cap/tab
	29	Seizure Disorder	Phenytoin	100 mg	cap/tab
	30	Ulcer	Ranitidine	150 mg	cap/tab
Supplementary list	31	Infectious disease	Amoxicillin	125mg/5ml	Suspension
	32	Infectious disease	Amoxicillin	250mg dispersible tablet	Dispersible tablet
	33	Infectious disease	Amoxicillin/Clavulanic acid	125mg+31.25mg/5ml	Suspension
	34	Infectious disease	Amoxicillin/Clavulanic acid	250mg + 125mg	Dispersible tablet
	35	Malaria	Chloroquine	150mg	Cap/tab
	36	Asthma	Beclomethasone	100mcg/dose	Inhaler
	37	Infectious disease	Benzyl penicillin	600mg = 1 million IU	Injection
	38	Seizure Disorder	Carbamazepine	100mg/5ml	Suspension
	39	Seizure Disorder	Carbamazepine	100mg	Chewable tablet
	40	Infectious disease	Ceftriaxone	500mg vial	Injection
	41	Infectious disease	Chloramphenicol	1 gram vial	Injection

	42	Infectious disease	Cotrimoxazole	100mg + 20mg<br>(also expressed as 400mg + 80mg)	Dispersible tablet
	43	Anxiety	Diazepam	2.5mg/ml	Rectal solution
	44	Anemia	Ferrous salt	30mg Fe/5ml	Suspension
	45	Infectious disease	Gentamycin	10mg/ml	Injection
	46	Pain/inflammation	Ibuprofen	200mg	Tablet
	47	Tuberculosis	Isoniazid	50mg	Scored tablet
	48	Pain	Morphine	10mg/5ml	Oral solution
	49	Pain	Morphine	10 mg	Immediate release tablet
	50	Dehydration	Oral rehydration solution (ORS)	To make 500ml	Sachet
	51	Dehydration	ORS	To make 1 liter	Sachet
	52	Seizure Disorder	Phenobarbital	200mg/ml	Injection
	53	Seizure Disorder	Phenobarbital	3mg/ml (also expressed as 15mg/5ml)	Oral liquid
	54	Seizure Disorder	Phenytoin	25 or 30mg/5ml	Suspension
	55	Seizure Disorder	Phenytoin	50mg	Chewable tablet
	56	Infectious disease	Procaine penicillin	1 gram = 1 million IU	Injection
	57	Xerophthalmia	Vitamin A	100,000IU	Capsules
	58	Dehydration	Zinc	20mg	Tablet (dispersible)
	59	Hypertension	Enalaril	5mg	Cap/tab
	60	Asthma (device)	Spacer for Inhalers	n/a	n/a

Table w2. Median price ratio for selected lowest priced generic medications, by sector

No.	Medicine Name	Lowest priced Generic Medication MPR [25th–75th percentile]			
		Public Sector	Retail Sector	Non-Profit Sector	Mixed Sector
**1**	Amitriptyline	N/A	13.3 [9.95-20.7]	N/A	N/A
**2**	Amlodipine	N/A	4.1 [2.4 - 5.8]	2.9 [1.4 - 4.1]	N/A
**3**	Amoxicillin	2.6 [2.2 - 3.5]	4.3 [3.5 - 4.3]	3.5 [2.2 - 4.3]	3 [2.6 - 3.3]
**4**	Amoxicillin suspension	2.8 [2.4 - 3.2]	3.2 [3.2 - 3.5]	2.5 [2.3 - 3]	2.3 [2.1 - 3.4]
**5**	Amoxicillin suspension 125 mg/5 ml	3.4 [2.8 - 4.2]	3.5 [3.2 - 4.6]	2.8 [2.8 - 3.5]	2.8 [2.8 - 3.8]
**6**	Atenolol	7.7 [5 - 12.2]	15.9 [10.6 - 26.5]	10 [5 - 23.2]	13.3 [7.3 - 26.5]
**7**	Atorvastatin	N/A	11.7 [11.4 - 11.7]	N/A	N/A
**8**	Azithromycin	1.9 [1.1 - 2.3]	3.8 [1.9 - 5]	0.6 [0.3 - 1.4]	1 [0.2 - 2.6]
**9**	Captopril	8.4 [2.9 - 10.5]	10.5 [7.1 - 10.5]	10.5 [4.2 - 18.1]	6.3 [2.1 - 10.5]
**10**	Ceftriaxone injection [1 g/vial]	2.6 [1.8 - 5.5]	5.5 [4.4 - 5.6]	4.4 [2.4 - 7.3]	3.7 [1.8 - 3.7]
**11**	Chloramphenicol Injection [1 g/vial]	N/A	3.1 [2.4 - 3.7]	N/A	N/A
**12**	Chloroquine	2.7 [2.7 - 5.3]	5.3 [5.3 - 7.3]	5.3 [2.7 - 5.6]	4 [2.7 - 6.6]
**13**	Ciprofloxacin	4.1 [2.5 - 4.5]	5.1 [4.2 - 8.2]	4.1 [3.5 - 4.1]	4.1 [1.7 - 4.1]
**14**	Clotrimazole topical cream	5.9 [4.9 - 8.6]	7.8 [6.7 - 9.3]	N/A	N/A
**15**	Co-trimoxazole suspension	3.6 [3 - 4.5]	4.5 [3.6 - 4.7]	3.6 [3 - 4.2]	3.3 [3 - 3.6]
**16**	Diazepam	20.7 [8.3 - 20.7]	20.7 [20.7 - 41.3]	16.5 [10.8 - 20.7]	8.3 [4.1 - 9.3]
**17**	Diclofenac	11.7 [8.1 - 16.1]	25.2 [14.7 - 29.3]	11.7 [10.3 - 26.4]	8.8 [5.9 - 13.9]
**18**	Enalapril	3.8 [2.6 - 5.7]	6.7 [5.9 - 8.4]	5.0 [2.7 - 6.7]	3.4 [3.4 - 5.9]
**19**	Enalapril [5 mg]	9.3 [7 - 11.6]	8.7 [7.4 - 12.4]	9.9 [9.3 - 11.1]	6.2 [6.2 - 7.7]
**20**	Fluoxetine	N/A	49.6 [48.7 - 82.6]	N/A	N/A
**21**	Furosemide	11.7 [5.9 - 13.9]	14.7 [11.7 - 17.6]	11.7 [11.7 - 12.9]	11.7 [7.7 - 11.7]
**22**	Glibenclamide	18.5 [13.6 - 4.1]	20.4 [14.8 - 35.2]	11.1 [7.4 - 31.5]	14.8 [7.4 - 18.5]
**23**	Hydrochlorothiazide	7.5 [6.8 - 20.4]	20.4 [13.6 - 38.1]	10.2 [6.8 - 13.6]	6.8 [6.8 - 8.5]
**24**	Ibuprofen [200 mg]	5.3 [3.7 - 8.5]	8.5 [8.5 - 16]	4.3 [4.3 - 8.5]	4.3 [4.3 - 8]
**25**	Ibuprofen [400 mg]	5.4 [2.7 - 5.4]	8 [5.7 - 9.4]	5.4 [3 - 7.4]	4 [2.7 - 5.4]
**26**	Metformin	10.2 [8.7 - 10.7]	11.8 [8.7 - 16.4]	N/A	N/A
**27**	Metronidazole	10.8 [8.1 - 15.8]	18 [10.8 - 18]	16.2 [7.2 - 18]	7.2 [6.3 - 10.8]
**28**	Omeprazole	4.2 [3.5 - 4.2]	4.2 [3.7 - 5.2]	3.8 [2.5 - 5.3]	2.9 [2.1 - 4.2]
**29**	Oral rehydration solution [1 L]	N/A	2.4 [1.5 - 4.4]	1.5 [1.5 - 2.9]	N/A
**30**	Paracetamol suspension [120 mg/5 ml or 125 mg/5 ml]	3.9 [3.2 - 4.9]	4.9 [3.2 - 6.46]	3.2 [3.2 - 4]	3.9 [3.2 - 5.1]
**31**	Phenytoin	N/A	30.2 [15.1 - 62.2]	N/A	N/A
**32**	Ranitidine	3.8 [2.5 - 6.3]	5.7 [3.8 - 6.3]	3.8 [3.8 - 15.1]	3.2 [2.5 - 3.5]
**33**	Salbutamol inhaler	2.1 [1.5 - 2.9]	2.3 [2.2 - 2.6]	1.7 [1.4 - 2.5]	2.2 [1.9 - 2.4]
**34**	Simvastatin	N/A	6.1 [5.1 - 12.1]	N/A	N/A
